# Combined effects of continuous exercise and intermittent active interruptions to prolonged sitting on postprandial glucose, insulin, and triglycerides in adults with obesity: a randomized crossover trial

**DOI:** 10.1186/s12966-020-01057-9

**Published:** 2020-12-14

**Authors:** Michael J. Wheeler, Daniel J. Green, Ester Cerin, Kathryn A. Ellis, Ilkka Heinonen, Jaye Lewis, Louise H. Naylor, Neale Cohen, Robyn Larsen, Paddy C. Dempsey, Bronwyn A. Kingwell, Neville Owen, David W. Dunstan

**Affiliations:** 1 Cardiovascular Research Group, School of Human Sciences (Exercise and Sport Science), The University of Western Australia, Perth, Australia; 2 Baker Heart and Diabetes Institute, 99 Commercial Rd, Melbourne, Victoria 3004 Australia; 3 Mary MacKillop Institute for Health Research, Australian Catholic University, Melbourne, Australia; 4 School of Public Health, The University of Hong Kong, Hong Kong, China; 5 Department of Psychiatry, University of Melbourne, Parkville, VIC Australia; 6 Turku PET Centre, University of Turku, Turku, Finland; 7 Rydberg Laboratory of Applied Sciences, ETN, Halmstad University, Halmstad, Sweden; 8 MRC Epidemiology Unit, Institute of Metabolic Science, University of Cambridge, Cambridge Biomedical Campus, Cambridge, UK; 9 Diabetes Research Centre, University of Leicester, Leicester General Hospital, Leicester, UK; 10 Centre for Urban Transitions, Swinburne University of Technology, Hawthorn, Australia

**Keywords:** Exercise, Sedentary behavior, Postprandial, Insulin resistance, Lipids, Glucose

## Abstract

**Background:**

Postprandial glucose, insulin, and triglyceride metabolism is impaired by prolonged sitting, but enhanced by exercise. The aim of this study was to assess the effects of a continuous exercise bout with and without intermittent active interruptions to prolonged sitting on postprandial glucose, insulin, and triglycerides.

**Methods:**

Sedentary adults who were overweight to obese (*n* = 67; mean age 67 yr SD ± 7; BMI 31.2 kg∙m^− 2^ SD ± 4.1), completed three conditions: SIT: uninterrupted sitting (8-h, control); EX+SIT: sitting (1-h), moderate-intensity walking (30-min), uninterrupted sitting (6.5-h); EX+BR: sitting (1-h), moderate-intensity walking (30- min), sitting interrupted every 30-min with 3-min of light-intensity walking (6.5 h). Participants consumed standardized breakfast and lunch meals and blood was sampled at 13 time-points.

**Results:**

When compared to SIT, EX+SIT increased total area under the curve (tAUC) for glucose by 2% [0.1–4.1%] and EX+BR by 3% [0.6–4.7%] (all *p* < 0.05). Compared to SIT, EX+SIT reduced insulin and insulin:glucose ratio tAUC by 18% [11–22%] and 21% [8–33%], respectively; and EX+BR reduced values by 25% [19–31%] and 28% [15–38%], respectively (all *p* < 0.001 vs SIT, all *p* < 0.05 EX+SIT-vs-EX+BR). Compared to SIT, EX+BR reduced triglyceride tAUC by 6% [1–10%] (*p* = 0.01 vs SIT), and compared to EX+SIT, EX+BR reduced this value by 5% [0.1–8.8%] (*p* = 0.047 vs EX+SIT). The magnitude of reduction in insulin tAUC from SIT-to-EX+BR was greater in those with increased basal insulin resistance. No reduction in triglyceride tAUC from SIT-to-EX+BR was apparent in those with high fasting triglycerides.

**Conclusions:**

Additional reductions in postprandial insulin-glucose dynamics and triglycerides may be achieved by combining exercise with breaks in sitting. Relative to uninterrupted sitting, this strategy may reduce postprandial insulin more in those with high basal insulin resistance, but those with high fasting triglycerides may be resistant to such intervention-induced reductions in triglycerides.

**Trial registration:**

Australia New Zealand Clinical Trials Registry (ACTRN12614000737639).

**Supplementary Information:**

The online version contains supplementary material available at 10.1186/s12966-020-01057-9.

## Background

Population ageing and rising obesity rates are placing people at increased risk of developing type 2 diabetes (T2D) and cardiovascular disease [1, 2]. While exercise can be an effective strategy for reducing key cardiovascular risk factors including postprandial glucose, insulin, and triglycerides [3, 4], it’s unclear how these benefits may be modified by sedentary behavior, which is highly prevalent among older adults. Objective measurements indicate that older adults (> 60 years) spend 65–80% of their waking day in sedentary behavior (i.e. sitting) [5]. It is possible that excessive time spent sedentary may attenuate some of the benefits of exercising [6].

Both total sedentary time and sedentary bout duration have been prospectively associated with increased mortality after adjustment for moderate-to-vigorous physical activity [7]. In experimental studies, prolonged periods of sitting induce greater postprandial rises in glucose, insulin, and triglycerides, relative to when sitting is interrupted with brief bouts of physical activity (typically 2–3 min every half hour) [8, 9]. Increases in these postprandial markers can promote lipid and glucose storage [10, 11], increase adhesion molecule expression and elevate oxidative stress [12], and have been prospectively associated with the development of T2D and cardiovascular disease [13–15]. Indeed, prolonged sitting is now recognized as a distinct behavioral consideration for postprandial markers of cardiometabolic risk [16].

To date, many experimental studies have compared the separate effects of continuous exercise, prolonged sitting, or regular interruptions to sitting on postprandial metabolic responses [17]. However, the combined effects of these behavioral approaches are also of interest since they often co-exist in a free-living setting. Homer et al. found that several hours of prolonged sitting followed by a 30-min bout of exercise in the afternoon, lowered postprandial insulin the following day during a period of prolonged sitting [18]. Interestingly, this effect was magnified when the exercise was combined with active interruptions to sitting. However, it is unclear whether the benefits of a morning bout of exercise are attenuated by subsequent exposure to prolonged sitting on the same day.

From a practical perspective, and in recognition of circadian variation in the metabolic responses to food intake and exercise [19, 20], there is a need for more research to investigate different patterns and combinations of prolonged sitting, interruptions to sitting, and continuous exercise on postprandial metabolism. In the current study, we examined the effect of a morning bout of moderate-intensity exercise with and without subsequent light-intensity interruptions to sitting, on postprandial glucose, insulin, and triglycerides in older adults who were overweight to obese. In addition, we performed secondary analyses to determine whether the degree of underlying insulin resistance and hyperlipidaemia predicted responses. We hypothesised that combining an exercise bout with active interruptions to prolonged sitting would lead to greater reductions in postprandial glucose, insulin, and triglycerides, than exercise following by prolonged sitting, or prolonged sitting with no exercise. In addition, we hypothesised that the magnitude of reduction in these postprandial markers would be predicted by the degree of underlying insulin resistance and fasting triglycerides.

## Methods

### Participants

**Fig. 1:**
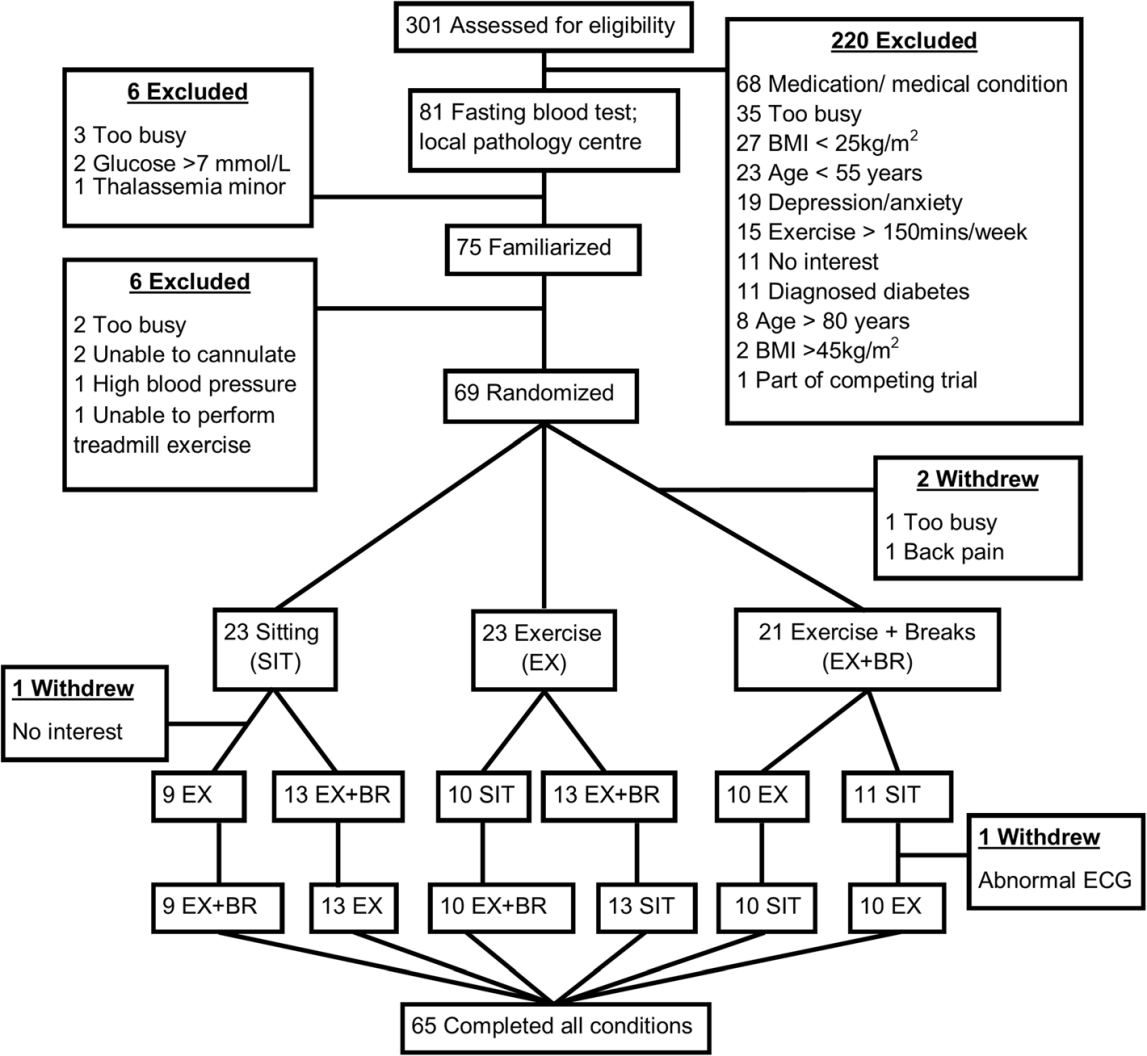
Consolidated Standards of Reporting Trials flow diagram

Men and postmenopausal women (≥55 to ≤80 years; body mass index ≥25 kg·m^− 2^ to < 45 kg·m^− 2^) were recruited from the local community and tested at two sites: the Physical Activity Laboratory, Baker Heart and Diabetes Institute, Melbourne, Australia; and, the Human Cardiovascular Exercise Research Laboratory, School of Human Sciences (Exercise and Sport Science), The University of Western Australia Perth, Australia. Ethical approval was obtained from The Alfred Hospital Ethics Committee (181–14) and The University of Western Australia Human Research Ethics Committee (RA/4/1/6990). Recruitment occurred between February 2015 and July 2017. Participants gave informed consent prior to taking part. The outcomes reported here are pre-specified secondary outcomes of a randomized crossover trial (ACTRN12614000737639), and the detailed methods, rationale, and design sections have been published independently [21]. The Consolidated Standards of Reporting Trials flow diagram (CONSORT) flow diagram is presented in Fig. 1, and the CONSORT checklist is provided as an additional file (see Additional File 1). The template for intervention description and replication (TIDieR) checklist is also provided (see Additional File 2). Full inclusion/exclusion criteria, and medication use, are provided as additional files (see Additional Files 3 and 4).

### Study design

Participants completed three conditions, in random order, with a washout period of 6 to 13 days between them: SIT: uninterrupted sitting (8-h, control); EX+SIT: sitting (1-h), moderate-intensity walking (30-min), uninterrupted sitting (6.5-h); EX+BR: sitting (1-h), moderate-intensity walking (30-min), sitting interrupted every 30-min with 3-min of light-intensity walking (6.5-h). A familiarization session was completed 3 to 5 days prior to testing, where participants were familiarized with treadmill walking. During the 48-h prior to testing, participants were instructed to avoid caffeine, alcohol and moderate-to-vigorous physical activity. Food was controlled from the night before testing where participants consumed a standardized dinner at home between 7 PM and 9 PM in place of their regular dinner. This meal was tailored for each participant to provide 33% of estimated daily energy requirements with a macronutrient profile of 55–58% carbohydrate, 29–31% fat and 12–15% protein as previously described [21].

### Exercise

The morning exercise performed in EX+SIT and EX+BR was a 30-min bout of moderate-intensity walking. The speed was set at 3.2 km∙h^− 1^ which was a walking pace for all participants, and the incline was tailored to induce a heart rate (HR) response indicative of moderate-intensity (HR between 65 and 75% of age predicted maximum HR). This incline was determined during the familiarization session. The three-minute light-intensity walking breaks were 3.2 km∙h^− 1^ with no incline for all participants. Heart rate (Polar Electro, Kempele, Finland) and ratings of perceived exertion (RPE scale 6–20; light intensity 9–11 RPE; moderate-intensity 12–15 RPE) were collected at 5-min intervals during the 30-min bout of exercise and at the end of each three-minute walking break. Participants were instructed to avoid getting out of the chair except to void, or to complete the predetermined treadmill walking in EX+SIT and EX+BR.

### Experimental day protocol

**Fig. 2:**
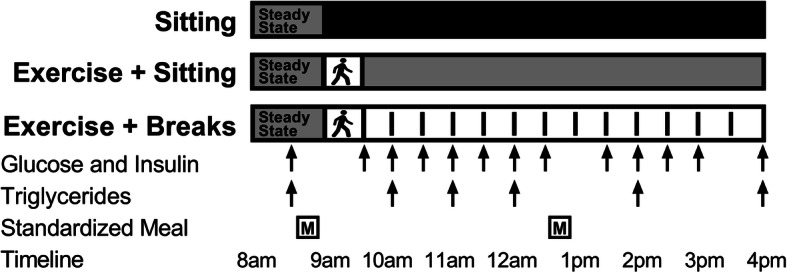
Experimental design. Participants completed three conditions in a random order separated by a minimum of six days: sitting (SIT): uninterrupted sitting (8-h, control); exercise+sitting (EX+SIT): sitting (1-h), moderate-intensity walking (30-min, denoted by walking figure) followed by uninterrupted sitting (6.5-h); exercise+breaks (EX+BR): sitting (1-h), moderate-intensity walking (30-min) followed by sitting (6.5-h) interrupted every 30-min with 3-min of light-intensity walking. Walking breaks are denoted by dashed lines in the EX+BR condition

Participants reported to the laboratory at ∼7 AM, following an overnight fast (> 10-h) and an indwelling cannula was inserted into an antecubital vein. The experiment began at ~ 8 AM with a 1-h steady state sitting period where a fasting blood sample was obtained prior to administration of a standardized breakfast meal; mean protein 30.3 g (SD 5.1); fat 25.6 g (SD 3.3); carbohydrate 109.1 g (SD 17.3). Lunch was consumed four hours after breakfast; mean protein 25.0 g (SD 2.6); fat 29.6 g (SD 3.4), carbohydrate 97.4 g (SD 19.7). Participants were allocated 20-min to consume breakfast and lunch, which were standardized in a manner identical to the standardized dinner. All meals remained the same for a given participant throughout the study. After breakfast, the protocol was followed according to randomization (Fig. 2).

### Biochemical analysis

Haematocrit and haemoglobin were determined from whole blood using a Beckman Coulter LH 785 according to standard methods, to calculate percent change in plasma volume from pre to post each condition. Coded samples were sent to an independent National Association of Testing Authorities/The Royal College of Pathologists of Australasia accredited laboratory on the day of testing for the determination of glucose and triglycerides. Blood was collected into fluoride/oxalate tubes for analysis of plasma glucose using the hexokinase method. Blood was collected into lithium heparin tubes for analysis of triglycerides using a COBAS Integra 400 + analyser (Roche Diagnostics, Indianapolis, IN). Serum was collected for the analysis of insulin. Serum samples coagulated for 1-h at room temperature (22–24 °C) prior to centrifuging at 2000 rpm (931 x g) for 15-min at 4 °C. Supernatants were removed and frozen immediately at − 20 °C and subsequently moved to a − 80 °C freezer at the end of the condition. Insulin was analysed from thawed samples using a chemiluminescent microparticle immunoassay (Architect ci16200; Abbott Diagnostics, Santa Clara, CA) with all samples from each participant analyzed in the same batch.

### Statistical analysis

Sample size calculations for this study were performed in relation to the pre-specified primary outcome of this trial (cognitive function), and the details of this have previously been reported [21]. The order of conditions was block-randomized and stratified by sex by an independent third party using a computer-generated random sequence and stored in sealed envelopes. Researchers were unblinded to the order of conditions when familiarization was complete, and participants were unblinded to the condition after the 1-h steady-state period for experimental visits 1 and 2. Postprandial glucose, insulin and triglycerides were summarized over the 8-h period as total area under the curve (tAUC) and positive incremental area under the curve (iAUC). Specifically, tAUC and iAUC represent the total area above a baseline of zero and the incremental area above a baseline of fasting, respectively, calculated using the trapezoidal method. Skewed variables were transformed by natural logarithm prior to analysis. Skewed variables are also presented in Table 1 as median and interquartile range, and normally distributed variables are presented as mean and standard deviation. Following recommendations on data analysis for cross-over trials [23], linear mixed models with random intercepts were used to test the effect of the condition on each outcome. A time-by-condition interaction term was included in regression models to examine the effect of time course on the conditions. The time course was plotted using back-transformed marginal means. Back-transformed marginal means were calculated by exponentiation of model output. Models were adjusted for potential confounders including age, sex, waist circumference, treatment order, testing site, baseline values and change in plasma volume calculated from haematocrit and haemoglobin using the Dill and Costill method [24]. The Homeostasis Model Assessment (HOMA2) was used to calculate surrogate indices of insulin resistance (HOMA2-IR) and β-cell function (HOMA2-%β*)* from fasting values of glucose and insulin on the morning of each condition*.* The average value across three conditions was obtained for HOMA2-IR, HOMA2-%β and fasting triglycerides, and these average values were examined as predictors of intervention-induced change in tAUC. Intervention-induced change in tAUC was calculated for each comparison as SIT minus EX+SIT, SIT minus EX+BR and EX+SIT minus EX+BR. Linear, quadratic and cubic relationships between each predictor variable (centered around its mean) and each dependent variable (i.e. intervention-induced change) were investigated using appropriate polynomial terms entered in mixed models, controlling for potential confounders. The best fitting polynomial equation was used to plot significant associations of the predictor variables with intervention-induced change in the dependent variables. A probability level of 0.05 was adopted. Statistical analyses were performed blinded to the study conditions using Stata 15 (StataCorp LP).

**Table 1:** Participant characteristics

Characteristic	(*n* = 67)
Sex (female/male)	35 / 32
Age (years)	67 ± 7
Body mass index (kg/m^2^)	31.2 ± 4.1
Waist circumference (cm)	105.4 ± 11.9
Impaired fasting glucose ^a^	11 (16%)
Fasting glucose (mmol/L) ^b^	5.2 ± 0.5
Fasting insulin (pmol/L) ^b^	51.6 [41.4–74.9]
HOMA2%β ^b^	105 ± 31
HOMA2-IR ^b^	1.3 ± 0.6
Hypertension ^c^	25 (37%)
Systolic blood pressure (mm Hg) ^b^	125 ± 14
Diastolic blood pressure (mm Hg) ^b^	74 ± 10
Resting heart rate (bpm) ^b^	62 ± 1
Fasting triglycerides (mmol/L) ^b^	1.2 [0.9–1.6]
Fasting total cholesterol (mmol/L) ^b^	5.2 ± 1.0
Fasting HDL-cholesterol (mmol/L) ^b^	1.3 ± 0.3
Fasting LDL-cholesterol (mmol/L) ^b^	3.3 ± 0.8

Data expressed as mean ± SD or n (%), fasting insulin and triglycerides are expressed as median [interquartile range]. ^a^ Impaired fasting glucose defined as 5.6 mmol/L to 6.9 mmol/L according to current guidelines [25]. ^b^ Average of 3 fasting measures across each condition. ^c^ hypertension defined as ≥130 mmHg systolic or ≥ 80 mmHg diastolic according to current guidelines [22]

## Results

A total of 301 individuals were telephone screened, 81 individuals were invited to have a blood test performed and 75 individuals were invited to take part in the familiarization session. Following familiarization, 69 participants were randomized. Due to dropout, 67 completed at least one condition and 65 completed all conditions. Analysis was performed on the 67 participants who completed at least one condition. Participants (35 females, 32 males) were older adults, 67 years (SD 7), and were overweight to obese, 31.1 kg∙m^− 2^ (SD 4.1). A small proportion (16%) were classified as having impaired fasting glucose (5.6 mmol∙L^− 1^ to 6.9 mmol∙L^− 1^) based on current recommendations [25]. Participant characteristics are displayed in Table 1.

### Exercise responses

The initial 30-min exercise bout induced identical HR and RPE responses between exercise conditions (mean ± SD); EX+SIT and EX+BR: 109 bpm (SD 12), 71%HRmax (SD 8), 11 RPE (SD 2). Average HR and RPE (6–20 scale) across all 12 walking breaks in EX+BR was 94 bpm (SD 2), 61%HRmax (SD 1) and 9 RPE (SD 0.4).

### Postprandial responses

**Fig. 3:**
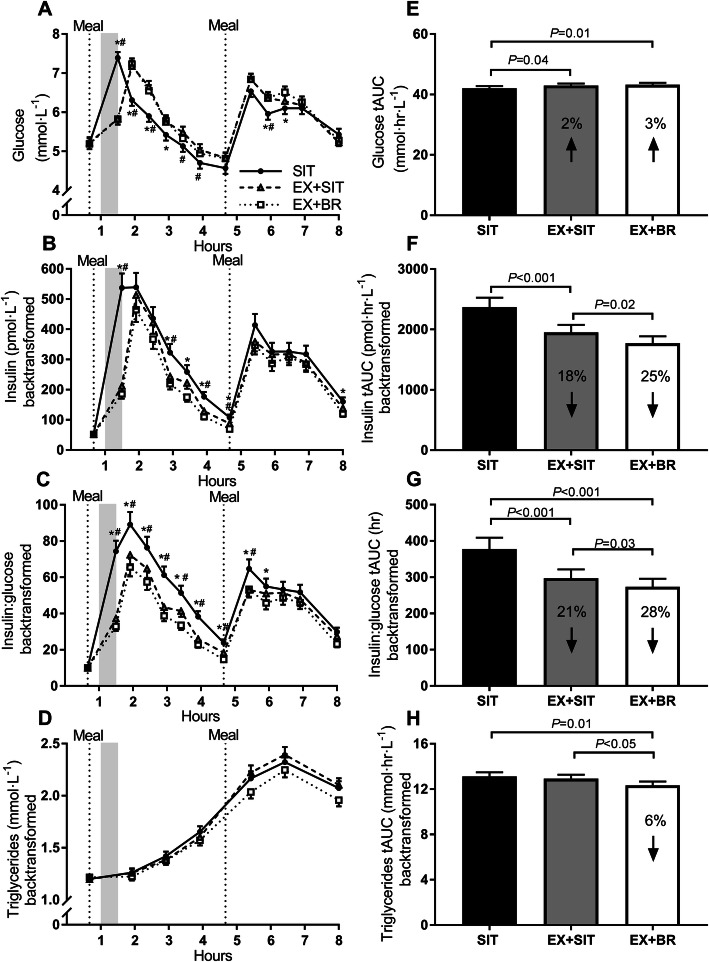
Postprandial glucose, insulin, insulin:glucose ratio and triglycerides. Panels **a**-**d** represent glucose, insulin, insulin:glucose ratio and triglycerides, respectively, displayed as a time course over 8-h. The shaded area represents the timing of the moderate-intensity exercise bout performed in exercise+sitting (EX+SIT) and exercise+breaks (EX+BR). Panels **e**-**h** represent glucose, insulin, insulin:glucose ratio and triglycerides, respectively, displayed as the total area under the curve (tAUC). For EX+SIT and EX+BR, the percentage change relative to sitting (SIT) is displayed within the bar. Data are marginal means and SEM, adjusted for age, sex, waist circumference, baseline values, change in plasma volume, testing site and treatment order. * *P* < 0.05 SIT vs EX+BR; # *P* < 0.05 SIT vs EX+SIT

**Fig. 4:**
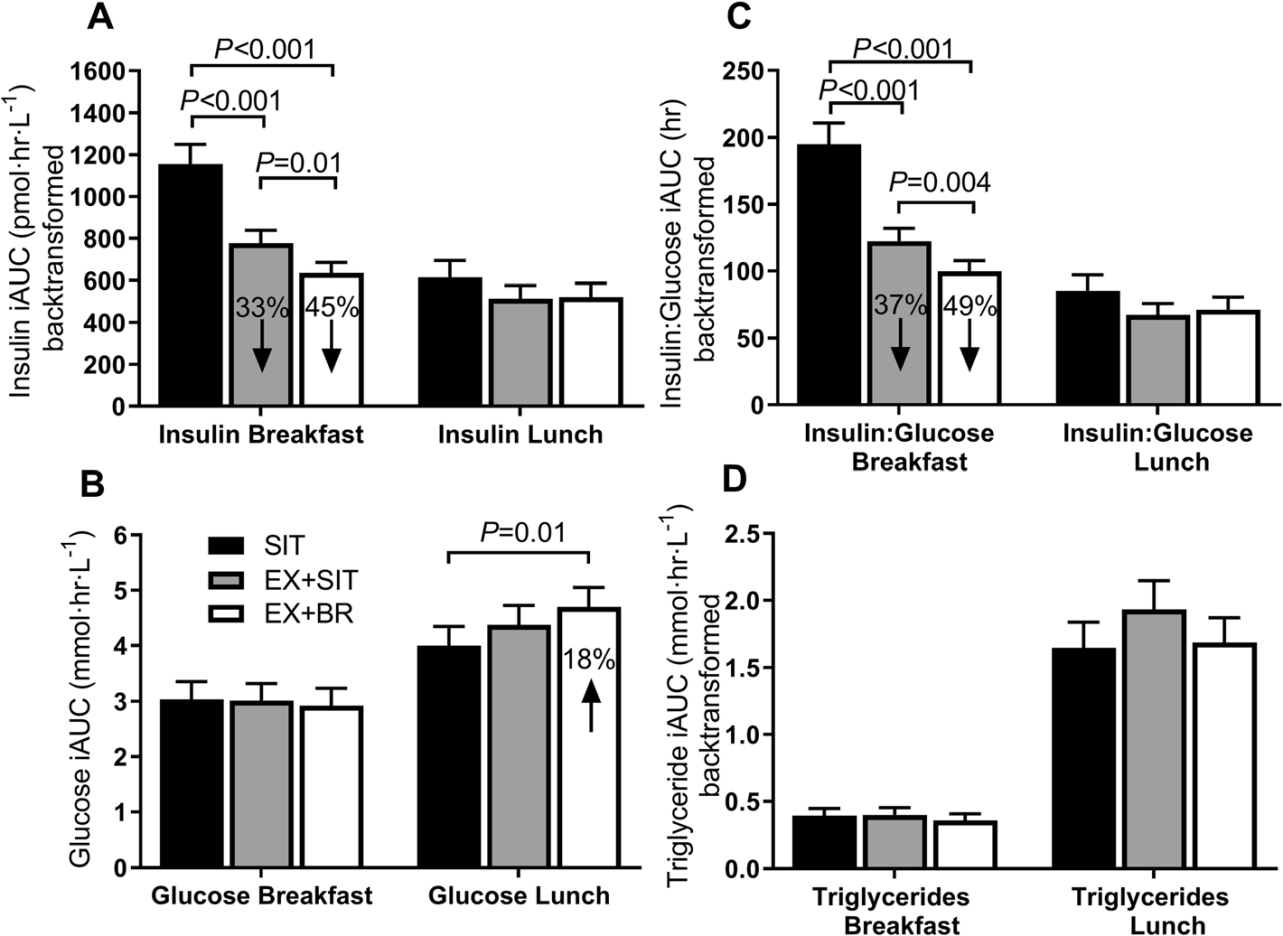
Meal-specific incremental area under the curve. Panels **a**-**d** represent the meal-specific insulin, glucose, insulin:glucose ratio, and triglyceride response, respectively, displayed as the positive incremental area under the curve (iAUC). The breakfast response represents the increase above the pre-breakfast time point until the pre-lunch time point. The lunch response represents the increase above the pre-lunch time point until the final time point. For EX+SIT and EX+BR, the change relative to sitting (SIT) is displayed within the bar. Data are marginal means and SEM, adjusted for age, sex, waist circumference, baseline values, change in plasma volume, testing site and treatment order

There was a small increase in glucose tAUC [95% CI] in EX+SIT 2% [0.1–4.1%, *p* = 0.04] and EX+BR 3% [0.6–4.7%, *p* = 0.01] relative to SIT (Fig. 3e). Relative to SIT, insulin tAUC (back-transformed; pmol·hr.·L^− 1^) was decreased by 18% [11–22%, *p* < 0.001] in EX+SIT and by 25% [19–31%, *p* < 0.001] in EX+BR. Insulin tAUC decreased by 9% [3–20%, *p* = 0.02] in EX+BR relative to EX+SIT (Fig. 3f). Relative to SIT, the insulin-to-glucose ratio (tAUC) decreased by 21% [8–33%, *p* < 0.001] in EX+SIT and by 28% [15–38%, *p* < 0.001] in EX+BR. Insulin-to-glucose ratio tAUC decreased by 8% [1–14%, *p* = 0.03] in EX+BR relative to EX+SIT (Fig. 3g). Triglyceride tAUC was reduced by 6% [1–10%, *p* = 0.01] in EX+BR relative to SIT and was reduced by 5% [0.1–8.8%, *p* = 0.047] in EX+BR relative to EX+SIT (Fig. 3h). Meal-specific responses are displayed as incremental area under the curve (Fig. 4).

### Predictors of intervention-induced change

**Fig. 5:**
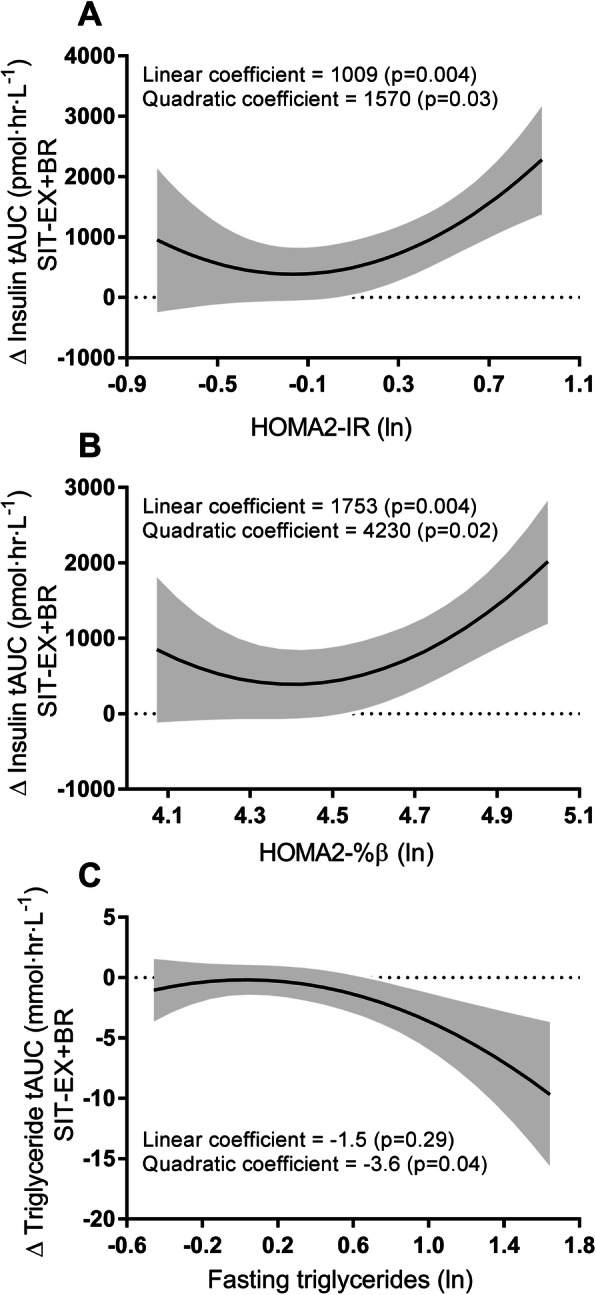
Associations between intervention-induced change and fasting baseline variables. Panels A-C represent the curvilinear relationships between intervention-induced change in insulin tAUC and HOMA2-IR, insulin tAUC and HOMA2-%β, triglyceride tAUC and fasting triglycerides, respectively. Fasting baseline variables were taken as the average across three conditions. The curved black line represents the line of best fit, calculated from a mixed model controlling for age, sex, waist circumference, change in plasma volume, testing site and treatment order. The shaded area represents the 95% confidence bands; ln indicates natural logarithm

Insulin resistance (HOMA2-IR) and beta cell function (HOMA2-%β) were both associated with intervention-induced change in insulin tAUC between SIT and EX+BR (Fig. 5a, b). These were positive quadratic (J-shaped) associations, suggesting that those with high basal insulin resistance or β-cell function had a larger magnitude of reduction in insulin tAUC between SIT and EX+BR. Fasting triglyceride concentration was associated with the magnitude of change in triglyceride tAUC between SIT and EX+BR (Fig. 5c). This was a negative quadratic association (inverted J-shape), suggesting that those with high fasting triglycerides were resistant to the intervention-induced reductions in triglyceride tAUC. None of the cubic regression coefficients were significant and differences in tAUC between SIT vs. EX+SIT or EX+SIT vs. EX+BR were not significantly associated to any of the baseline measures.

## Discussion

This study demonstrates that post-exercise reductions in postprandial insulin and triglycerides are amplified by the addition of physically-active interruptions to prolonged sitting. This represents an opportunity to optimize current exercise strategies to reduce the risk of cardiovascular disease and T2D in older adults who are overweight to obese.

### Postprandial glucose and insulin responses

Previous studies in adults have demonstrated reduced postprandial glucose and insulin levels when sitting is interrupted with intermittent bouts of light-intensity activity [17]. In 19 middle-aged adults (mean ± SD age 53.8 ± 4.9 years; BMI: 31.2 ± 4.1 kg/m^2^) who were physically inactive and sedentary, our group observed reductions of approximately 25 and 23% in the 5-h iAUC for glucose and insulin, respectively, when sitting was interrupted with short light-intensity walking breaks every 30-min [26]. In 70 adults (mean ± SD 25.9 ± 5.3 years; BMI: 23.6 ± 4.0 kg/m^2^) who were physically inactive, Peddie et al. demonstrated an approximate 39, and 26% reduction in the 9-h iAUC for glucose and insulin, respectively, when sitting was interrupted with short moderate-intensity walking breaks every 30-min [27]. In the current study of older adults who were overweight to obese, small increases in glucose tAUC of 2 and 3% were observed during EX+SIT and EX+BR, respectively. While the intermittent light-intensity activity breaks in the current study could be expected to reduce postprandial glucose due to contraction-mediated glucose uptake [28], the antecedent bout of moderate intensity exercise would be expected to increase hepatic glucose production even in the postprandial state [29, 30].

Similar to the current study, Peddie et al. also included a condition where a 30-min bout of exercise was followed by prolonged sitting [27]. Interestingly, a small increase in the glucose time course during the exercise period was observed [27]. However, this increase was not sustained and the exercise condition resulted in a non-significant decline of ~ 3% in the 9-h glucose iAUC compared to prolonged sitting [27]. Key differences with the current study population are that Peddie et al. included younger participants with a healthier metabolic profile. Other studies that have observed sustained but non-significant increases in glucose following a single exercise bout have included participants who were older, overweight, or with impaired fasting glucose [31–33]. This may partly explain why we observed small but sustained increases in glucose following exercise in our population of older adults, who were overweight/obese, and where some (16%) had impaired fasting glucose.

The observed increases in glucose in the current study equate to an approximate 0.2 mmol∙L^− 1^ increase in average glucose over 8-h in the exercise conditions relative to SIT. Such increases are important for muscle substrate provision, but unlikely to have adverse clinical consequences [34]. These small increases in glucose should be considered alongside the concurrent and large decreases in insulin tAUC of 18 and 25% during EX+SIT and EX+BR, respectively. Our meal-specific analysis also revealed an interesting pattern whereby the large reductions in insulin occurred mostly following morning exercise in the post-breakfast period, whereas the small increases in glucose seemed to occur in the afternoon following the lunch meal. Perhaps the post-breakfast exercise leading to rapid and substantial declines in insulin initiated a counter-regulatory response that facilitated hepatic glucose production during the post-lunch period. Indeed, a fall in insulin can sensitize the liver to glucagon [35]; and postprandial glucagon secretion has been observed in middle-aged participants who were obese but otherwise healthy [36]. Therefore, a fall in insulin followed by a postprandial rise in glucagon may work synergistically to increase hepatic glucose output [37]. In a recent meta-analysis, counter-regulation of glucose following continuous exercise has been suggested to explain why continuous exercise is inferior to isoenergetic intermittent exercise in terms of postprandial glucose lowering [38].

Although the outcomes of the current study cannot explain the mechanisms underlying changes in glucose and insulin dynamics, the overall insulin-to-glucose ratio tAUC was decreased by 21 and 28% during EX+SIT and EX+BR, respectively, compared to SIT. Since the difference between EX+SIT and EX+BR was also significant, this suggests the additional benefit of combining exercise with subsequent breaks in sitting is an approximate 7% reduction in the insulin-to-glucose ratio tAUC. These reductions likely translate to a reduced demand on insulin secretion. Our data suggest that a bout of exercise may curb the compensatory hyperinsulinemia which is associated with progression of insulin resistance by reducing demand for insulin secretion, and this effect is enhanced with the addition of intermittent breaks in sitting.

### Postprandial triglyceride responses

An interesting finding from the current study was that the postprandial triglyceride tAUC was reduced by 6% in EX+BR, relative to SIT. Since this reduction was also significantly lower than EX+SIT, it suggests that there may be a synergistic effect of combining an exercise bout with active interruptions to sitting on lowering same-day postprandial triglyceride responses. This may help mitigate the development of insulin resistance since hyperlipidemia promotes the storage of lipids in muscle, liver, and visceral adipose tissue [11, 39].

In contrast to some previous studies, we observed a reduction in postprandial triglycerides during the 8-h intervention. In a recent meta-analysis of experimental studies comparing prolonged sitting to sitting interrupted with light to moderate-intensity activity, postprandial triglycerides were only reduced 12–16 h after the interventions had ended [8]. Similarly, one previous study demonstrated that regular interruptions to sitting followed by an exercise bout at the end of the day reduced postprandial triglycerides the next day [18]. The suggested reason for this delayed effect is exercise-induced activation of lipoprotein lipase (LPL) which can increase for up to 24-h post-exercise [40]. In the postprandial state, LPL plays an important role in clearing triglycerides from circulation by liberating free fatty acids from triglyceride-rich lipoproteins [41].

Similar to the current study, Miyashiata et al. observed a same day reduction of approximately 11% in the 8-h triglyceride tAUC [32]. Interestingly, this occurred in response to the intermittent light-intensity walking breaks condition and not the continuous exercise plus prolonged sitting condition. This suggests that intermittent exercise may be more effective at lowering same day triglycerides than continuous exercise, a concept supported by our finding that no significant reduction in triglyceride tAUC was observed in EX+SIT relative to SIT. Without a fourth condition (sitting interruptions without exercise), the question remains whether breaks in sitting alone would have been sufficient to lower the triglyceride tAUC in the current study population.

There is reason to suspect that combining exercise with breaks in sitting had a synergistic effect on lowering triglycerides in the current study. This is because the initial exercise bout substantially lowered insulin levels. Since insulin can stimulate LPL activity in adipose tissue while inhibiting activity in skeletal muscle [42], large declines in insulin may have facilitated a shift in the site of triglyceride clearance away from adipose tissue and towards the working muscles. A higher blood flow to the lower limb working muscles would also be expected when exercise is followed by intermittent breaks in sitting. Indeed, we have previously demonstrated that intermittent activity breaks can increase femoral artery blood flow by an average of 46% over 5 h, relative to uninterrupted sitting [43]. The reduction in triglycerides may be a result of directing fatty acids to the lower limb working muscles, which can act as a ‘sink’ for triglycerides due to activity of lipases and subsequent fatty acid utilization for energy production [44]. Alternatively, the reduction in circulating triglycerides could be explained in the context of reduced insulin, as insulin can inhibit the production of dietary chylomicrons [45]. Ultimately, it is unclear from the current study what proportion of the reduction in triglycerides during EX+BR relates to an increased clearance, versus a decreased production of lipoproteins, and this should be investigated in future experiments.

### Baseline measures as predictors of intervention-induced change

While the precise mechanism of the observed reduction in postprandial triglycerides is unknown, our analyses offered insight on what may predict the magnitude of reduction. We observed a curvilinear relationship suggesting that those with a high fasting triglyceride level (above 0.6 ln or ~ 1.8 mmol∙L^− 1^) at baseline may be resistant to the intervention-induced decreases in postprandial triglycerides observed overall in EX+BR compared to SIT. A higher fasting triglyceride level could indicate hepatic insulin resistance, which would promote hepatic triglyceride production [30]. In addition, insulin resistance has been associated with increased production of intestinal lipoproteins in the postprandial state [46], and has also been associated with decreased muscle lipoprotein lipase activity [47]. This may partly explain the current findings, although HOMA2-IR was not significantly associated with intervention-induced change in postprandial triglycerides (data not shown). However, HOMA2-IR is not the gold standard method to assess insulin resistance. Future studies that more accurately measure insulin resistance are needed to corroborate the current finding. One alternative is the oral glucose minimal model technique with a tracer which measures the selective effect of insulin on glucose disposal under physiological conditions [48].

Similar to previous research [49], we observed that those with higher baseline HOMA2-IR and HOMA2-%β had larger reductions in postprandial insulin when comparing the response during EX+BR to the response during SIT. However, our analyses indicate that this may be a curvilinear relationship. This suggests there may be a threshold past which greater underlying insulin resistance or β-cell function is exponentially associated with greater intervention-induced reductions in postprandial insulin.

### Strengths and limitations

Strengths of our study include the large sample size and randomized crossover design, where participants serve as their own controls. In addition, food intake was controlled from the evening before each experimental condition and on the day of each experiment with the provision of standardized mixed meals. The use of real food mixed meals also represents a more ecologically valid nutrient stimulus compared to liquid meals. Another strength is the assessment of both linear and non-linear relationships between intervention-induced change and their predictors within a mixed model controlling for potential confounders. Since the relationships were found to be non-linear, this aids the interpretation suggesting that the observed relationships with intervention-induced change are only present when the value of the predictor is past a certain threshold.

There are also some limitations to consider. For example, it is unknown whether the benefits observed during EX+BR relative to EX+SIT are due to increased energy expenditure and/or muscle activation of the walking breaks or due to their intermittent nature. This mechanistic question may be particularly important in relation to postprandial triglycerides, as the role of muscle LPL activity is less clear in acute exercise studies of a shorter duration (≤8 h) [8, 50]. Future studies will need to tease apart the role of energy expenditure, muscle activation, and timing of activity breaks in order to optimize strategies to reduce postprandial triglycerides. In addition, these results cannot necessarily be extrapolated to younger, leaner participants. Although we had a large sample size, our participants did not span a broad variation of metabolic phenotypes (i.e. dysglycemia and dyslipidemia). Thus, our analyses of predictors of intervention-induced change offer insights on associations only within the range of glycaemia and lipidaemia exhibited by the current study population. Future studies employing similar analysis techniques would benefit from including participants across a wider spectrum of health and disease. In addition, future studies are required to determine whether the effects observed in the acute setting are sustained when such strategies are repeated over longer periods of time.

## Conclusions

In conclusion, we have demonstrated combined effects of continuous exercise and intermittent active interruptions to sitting on reducing postprandial insulin, insulin-to-glucose ratio and triglycerides. These results are informative from a practical perspective, since it is possible for a person to achieve the guideline-recommended level of daily exercise, while also sitting for prolonged periods in the same day (i.e. the active commuter with a sedentary job). In addition, we observed that beyond a certain threshold, those with a greater degree of underlying insulin resistance or β-cell function had greater intervention-induced reductions in postprandial insulin. We also observed that those past a threshold of high fasting triglycerides were resistant to the overall intervention-induced reductions in postprandial triglycerides.

Taken together, these findings offer a unique practical insight into the combined effects of exercise and sedentary behavior on postprandial markers of cardiometabolic risk, and highlight clear areas for future investigation. These insights may assist in optimizing future strategies to reduce the risk of cardiovascular disease and T2D in older adults who are overweight to obese.

## Supplementary Information

**Additional file 1.** CONSORT Checklist. Completed CONSORT checklist.

**Additional file 2.** TIDieR checklist. Completed TIDieR checklist.

**Additional file 3: Table S1**. Full inclusion and exclusion criteria.

**Additional file 4: Table 2**. Participant concomitant medications.

## Data Availability

The data that support the findings of this study are available from the corresponding author upon reasonable request.

## Abbreviations

EX+BR: Exercise plus breaks condition
EX+SIT: Exercise plus sitting condition
HOMA2-%β: Homeostasis model assessment of beta cell function
HOMA2-IR: Homeostasis model assessment of insulin resistance
HR: Heart rate
iAUC: incremental area under the curve
LPL: Lipoprotein lipase
RPE: Rating of perceived exertion
SD: Standard deviation
SIT: Sitting condition
tAUC: total area under the curve
T2D: Type two diabetes
