# Supplementary material for: Combined effects of continuous exercise and intermittent active interruptions to prolonged sitting on postprandial glucose, insulin, and triglycerides in adults with obesity: a randomized crossover trial
Source: Int J Behav Nutr Phys Act. 2020 Dec 14;17:152. doi: 10.1186/s12966-020-01057-9 (PMC7734727; doi:10.1186/s12966-020-01057-9)
Supplement: Supplementary file 3 — Additional file 3: Table S1. Full inclusion and exclusion criteria. [file 12966_2020_1057_MOESM3_ESM.docx]

**Additional table 1.** Inclusion and exclusion criteria.

| Inclusion criteria |
| --- |
| Men and post-menopausal women |
| ≥55 to ≤80 years of age |
| BMI ≥25 kg/m^2^ to <45 kg/m^2^ |
| English speaking |
| Exclusion criteria |
| Self-reported sitting < 5 hours per day |
| Self-reported MVPA ≥150 min/week for > 3 months |
| Cognitive impairment (MMSE score <24) |
| Probable dementia (TICS score of <19) |
| Depressive symptoms (GDS score >6 or HADS-D score >8) |
| Diagnosed diabetes |
| Beta blockers, lipid lowering, anti-anxiety or antidepressant medication |
| Excessive alcohol consumption (AUDIT score >8) |
| Abnormal resting ECG (determined by study doctor) |
| High blood pressure (systolic > 160 mmHg or diastolic>100 mmHg) |
| Exercise limiting illness or physical problem |

BMI, body mass index; ECG, electrocardiogram; GDS, Geriatric Depression Scale; HADS-D, Hospital Anxiety and Depression Scale – depression subscale; MMSE, Mini Mental State Exam; MVPA, moderate-to-vigorous physical activity; TICS, Telephone Interview of Cognitive Status.
