# Supplementary material for: Combined effects of continuous exercise and intermittent active interruptions to prolonged sitting on postprandial glucose, insulin, and triglycerides in adults with obesity: a randomized crossover trial
Source: Int J Behav Nutr Phys Act. 2020 Dec 14;17:152. doi: 10.1186/s12966-020-01057-9 (PMC7734727; doi:10.1186/s12966-020-01057-9)
Supplement: Supplementary file 4 — Additional file 4: Table 2. Participant concomitant medications. [file 12966_2020_1057_MOESM4_ESM.docx]

Additional table 2. Participant concomitant medications.

| Medication | *N* (%) |
| --- | --- |
| Angiotensin II receptor blockers | 12 (18%) |
| Calcium channel blockers | 8 (12%) |
| Angiotensin converting enzyme inhibitors | 5 (7%) |
| Proton pump inhibitor | 4 (6%) |
| Non-steroidal anti-inflammatory | 4 (6%) |
| Thyroxine | 3 (4%) |
| Diuretic | 2 (3%) |
| Bronchodilator | 3 (4%) |
| 5-alpha reductase enzyme inhibitor | 2 (3%) |
| Alpha-blocker | 2 (3%) |
| Hormonal replacement therapy | 1 (1%) |
| Anticonvulsant | 1 (1%) |
| Inhaled corticosteroid | 1 (1%) |
